# Marine-Derived Antioxidants: A Comprehensive Review of Their Therapeutic Potential in Oxidative Stress-Associated Diseases

**DOI:** 10.3390/md23060223

**Published:** 2025-05-22

**Authors:** Ruiqiu Zhang, Yuke Ren, Tianqi Ren, Yue Yu, Bo Li, Xiaobing Zhou

**Affiliations:** 1 National Institutes for Food and Drug Control, Chinese Academy of Medical Sciences and Peking Union Medical College, Beijing 100730, China; ruiqiuzhang0925@163.com (R.Z.);; 2 National Center for Safety Evaluation of Drugs, National Institutes for Food and Drug Control, Beijing 100176, China; 3 School of Basic Medicine and Clinical Pharmacy, China Pharmaceutical University, Nanjing 210009, China

**Keywords:** marine antioxidants, oxidative stress diseases, bioactive marine compounds, antioxidant activity of marine organisms

## Abstract

Oxidative stress is a critical factor contributing to the pathogenesis of numerous diseases, including cardiovascular disorders, diabetes, and neurodegenerative conditions. In recent years, marine-derived antioxidants have emerged as promising therapeutic agents due to their unique biological activities and diverse sources. This comprehensive review explores the therapeutic potential of various marine antioxidants in mitigating oxidative stress-associated diseases. Marine organisms are rich in bioactive compounds, such as polysaccharides, polyphenols, carotenoids, peptides, and vitamins, which exhibit potent antioxidant and free radical scavenging abilities. These compounds have been shown to effectively inhibit oxidative reactions, repair oxidative damage, and enhance the body’s antioxidant defense mechanisms. For instance, marine polysaccharides and their derivatives can scavenge reactive oxygen species (ROS), protect neurons from oxidative damage, and alleviate inflammation in neurodegenerative diseases like Alzheimer’s and Parkinson’s diseases. Similarly, marine unsaturated fatty acids, such as omega-3 polyunsaturated fatty acids (PUFAs), have been found to reduce cardiovascular risks by lowering serum triglyceride levels and improving vascular endothelial function. Additionally, marine-derived superoxide dismutase (SOD) plays a crucial role in neutralizing ROS, thereby offering protection against oxidative stress in various diseases. Despite these promising findings, challenges remain in the field, including the need for improved extraction and purification technologies, more comprehensive activity evaluation systems, and further research into the safety and bioavailability of these compounds. This review provides a detailed overview of the current research status, highlighting the types, structural characteristics, antioxidant activities, and mechanisms of action of marine antioxidants. It also identifies key areas for future research and development, aiming to harness the full potential of marine-derived antioxidants in the prevention and treatment of oxidative stress-related diseases.

## 1. Introduction

Marine organisms have been widely recognized as rich producers of bioactive chemicals with medicinal promise [1,2]. Various substances obtained from marine species have attracted interest due to their wide range of biological functions, such as anti-inflammatory and antioxidant properties [1,3]. Marine microorganisms have a tremendous ability to sense and respond to their surroundings and can survive in stressful environments by producing different molecules. Since oxidative stress is directly or indirectly involved in various pathological conditions in humans, we believe that marine-derived antioxidant molecules will have broad prospects as new types of antioxidant molecules.

In today’s medical and health field, oxidative stress diseases have become a major challenge that troubles humanity. With the acceleration of industrialization and the change of lifestyle, cardiovascular diseases, neurodegenerative diseases, diabetes and its complications, cancer, skin oxidative damage, and other problems are increasingly common [4,5,6]. At the same time, scientists have turned their attention to the vast and boundless ocean, exploring new therapeutic approaches, and ocean-derived antioxidants have emerged. The ocean on Earth occupies about 71% of the surface area. Over the long course of evolution, marine organisms have evolved unique compounds to adapt to extreme and complex environments, such as high-pressure, low-temperature, and high-salinity environments. These compounds contain excellent antioxidant properties, making them potential remedies for combating oxidative stress diseases. For example, microorganisms around deep-sea hot springs develop high-temperature-resistant superoxide dismutase (SOD) in high-temperature and -pressure environments [7]. Brown algae survive in the intertidal zone of the ocean, where tides surge and light changes, accumulating strong antioxidant substances such as fucoxanthin and fucoidan [4,8]. They are expected to break through the limitations of traditional treatment methods, bring new hope to patients with various oxidative stress diseases, and start a health defense war originating from the ocean.

Active free radicals can cause a range of damage to biological membranes and other tissues. There are many substances in animal, plant, and microbial tissues that can act as antioxidants [9,10]. These substances are mainly free radical scavengers and antioxidants. Free radical scavengers and antioxidants mainly demonstrate their activity by directly clearing free radicals or promoting the enhancement or recovery of the body’s own antioxidant defense system [11,12]. In recent years, with the in-depth development of marine biology research and the improvement of research technology, compounds with antioxidant activity have been continuously discovered from marine organisms [13,14]. Marine-derived antioxidants have shown great potential in treating oxidative stress-related diseases due to their unique biological activity and wide range of sources [14,15]. Oxidative stress is a common pathological mechanism of many diseases, such as cardiovascular diseases, diabetes, and neurodegenerative diseases. It is characterized by an imbalance between reactive oxygen species (ROS) production and endogenous antioxidant defenses [16,17], serving as a central pathological mechanism underlying numerous chronic diseases. While terrestrial antioxidants have been extensively characterized, the marine ecosystem—harboring 80% of Earth’s biodiversity—represents an underexplored reservoir of unique antioxidative compounds. These marine-derived molecules exhibit distinct structural features and enhanced ROS scavenging capacities compared to their terrestrial counterparts, attributed to evolutionary adaptations to extreme marine environments. While terrestrial sources of antioxidants (e.g., polyphenols from plants) have been extensively studied, the marine environment—a treasure trove of biodiversity—offers unique bioactive compounds with potent antioxidant properties. Marine organisms are rich in various antioxidant substances, such as polysaccharides, polyphenols, carotenoids, and peptides, and they have significant free radical scavenging abilities and antioxidant activity. Research has shown that these marine antioxidants can effectively inhibit oxidative reactions and repair oxidative damage, thus demonstrating great potential for application in the prevention and treatment of oxidative stress-related diseases [18,19]. Moreover, they can clear endogenous and exogenous free radicals through various pathways, with strong antioxidant effects, diverse types, complex structures, and low side effects [20].

However, there are still some shortcomings and challenges in the research in this field. Firstly, the extraction and purification technology of marine antioxidants is relatively underdeveloped, and the lack of equipment and methods leads to low extraction efficiency and low purity [21]. Secondly, the activity evaluation system is not yet perfect, and there are differences between existing in vitro and in vivo experiments. Moreover, the evaluation indicators cannot fully reflect the actual effects of antioxidants in vivo.

Marine organisms, ranging from macroalgae to deep-sea fish, invertebrates, and microorganisms, synthesize diverse antioxidants to survive extreme conditions such as high salinity, UV radiation, and oxidative pressure [1,2,22]. These adaptations have led to the evolution of structurally unique molecules, including sulfated polysaccharides, omega-3 PUFAs, and metalloenzymes like SOD. For instance, fucoidan—a sulfated polysaccharide from brown algae—exhibits ROS scavenging activity 2–3 times higher than its terrestrial counterparts due to its sulfate-rich backbone.

Despite their therapeutic promise, marine antioxidants face challenges in clinical translation. Extraction and purification technologies remain underdeveloped, with traditional methods often yielding low-purity compounds. For example, only 30–40% of crude fucoidan extracts retain bioactivity after conventional ethanol precipitation. Furthermore, the structural complexity (e.g., branching patterns in polysaccharides) complicates structure–activity relationship (SAR) studies [23,24]. Recent advances in omics technologies and bioinformatics tools are shedding light on how specific structural motifs drive antioxidant efficacy. For instance, the 1,3-linked α-L-fucose residues in fucoidan are critical for binding free radicals, while sulfate groups at the C-2 and C-4 positions enhance hydrophilicity and ROS neutralization.

This review systematically summarizes and generalizes the research achievements in this field in recent years, including the types, structural characteristics, antioxidant activity, and mechanisms of action of antioxidants from different marine biological sources, providing researchers with a comprehensive overview of the current research status, helping them quickly understand the latest developments and major findings in the field. We also address current limitations—such as the bioavailability issues with oral SOD—and propose future directions, including nanotechnology-based delivery systems and clustered regularly interspaced short palindromic repeat (CRISPR)-engineered marine microbes for sustainable production. By bridging the gaps between marine biology, pharmacology, and material science, this work aims to improve people’s understanding of marine antioxidants and their application in oxidative stress diseases. At the same time, based on the summary of existing research, this review deeply analyzes the current shortcomings and challenges in this field, such as the technical difficulties in extracting and purifying marine antioxidants, the imperfect activity evaluation system, the insufficient safety and toxicology research, and the stability and cost issues in application development. By clarifying these issues, it can provide direction for future research, encourage researchers to conduct in-depth research on these shortcomings, and promote further development of marine antioxidant research.

## 2. Sources of Marine-Derived Antioxidants

### 2.1. Polysaccharides from Algae

**Figure 1:**
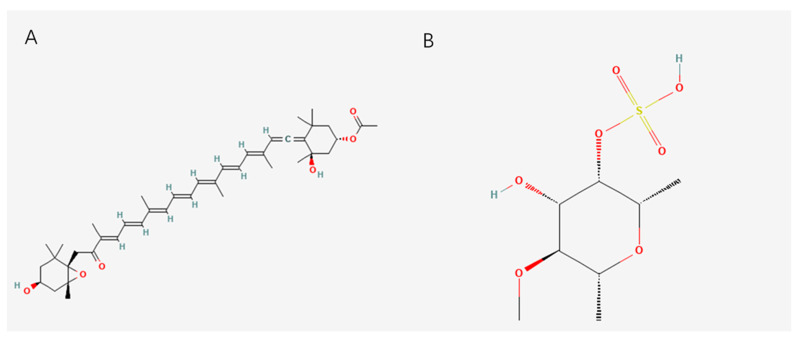
Structure diagram of (**A**) fucoxanthin and (**B**) fucoidan.

Algae are one of the most studied and promising sources of marine antioxidants, and they are not only diverse but are also rich in a variety of bioactive substances with antioxidant properties. Brown algae, including kelp, wakame, and laminaria, are widely distributed in the ocean. The antioxidants contained in them are fucoxanthin and fucoidan, whose structures are shown in Figure 1. Fucoxanthin is a carotenoid mainly found in brown algae, and it has a variety of biological activities, especially a strong antioxidant capacity [25]. It can effectively remove free radicals and reduce oxidative stress, thus protecting cells from damage [26]. Its antioxidant mechanism may be related to its enhancement of antioxidant enzyme activity. In addition, fucoxanthin also has anti-inflammatory, anti-cancer, and other effects. Fucoxanthin has shown protective effects against oxidative damage in different disease models, such as myocardial fibrosis, non-alcoholic fatty liver disease, and renal ischemia–reperfusion injury, and it has been shown to protect tissues by reducing oxidative stress and inflammatory responses [27,28,29].

Fucoidan is a sulfated polysaccharide extracted from brown algae [30]. Its structure is based on α—L-fucose, which is linked by α (1 → 3) and α (1 → 4) glycosidic bonds [31,32]. The sulfated group is modified at the C2 or C4 position of fucose, and the sulfated degree is 15%~40% [33]. It is the key determinant of activity. It has direct antioxidant effects, such as free radical scavenging and metal ion chelation, while activating endogenous antioxidant systems, such as the Nrf2/ARE pathway, and inhibiting the oxidative stress–inflammation cycle, such as the NF-κB pathway. Its structural characteristics are closely related to its antioxidant activity, and high sulfation, a low molecular weight, glucuronic acid branching, and the α (1→3) main chain ratio can significantly enhance its antioxidant capacity [34].

### 2.2. Polysaccharides from Animals

Animal polysaccharides mainly include chitosan and glycosaminoglycan. Chitosan is a biopolymer derived from the shells found in the exoskeletons of crustaceans such as shrimp and crabs [35,36]. Because of its biocompatibility, biodegradability, and non-toxicity, it has received wide attention in various fields [37,38]. Chitosan has been investigated for its potential role in the management and treatment of neurodegenerative diseases by scavenging free radicals and reducing oxidative stress [39,40]. Chitosan oligosaccharides can reduce Cu^2+^-induced oxidative damage and apoptosis involved in Nrf2 activation [40], and chitosan can help reduce oxidative damage and can be used as a nutritional agent for Alzheimer’s disease (AD) treatment. Additionally, both chitosan and its quaternary derivative N-trimethyl chitosan chloride exhibit concentration-dependent inhibiting activity on Aβ 40 fibrillogenesis, mainly depending on the attractive electrostatic interactions between the positively charged moieties in chitosan and the negatively charged residues in Aβ40 [41]. This effect may be related to the occurrence and development of AD.

**Figure 2:**
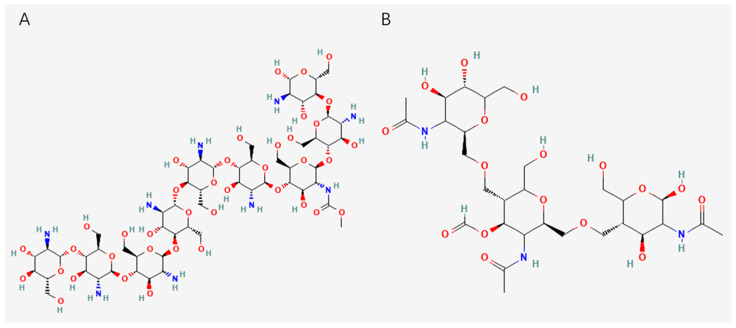
Structure diagram of (**A**) chitosan and (**B**) chitin.

Besides chitosan, chitin is the second most abundant natural polymer in the world after cellulose and is synthesized by a large number of organisms to form exoskeletons [42,43]. It can be divided into three different groups: alpha-chitin, which is typically extracted from the exoskeletons of crustaceans; beta-chitin, which is extracted from squid pens; and gamma-chitin, which is extracted from fungi and yeast [44]. Chitin is inelastic and strong, and once it reacts with calcium carbonate (CaCO_3_), it forms a more robust structure, like the exoskeletons of mollusks and crustaceans [45]. However, due to its acetyl group, it is also hydrophobic and insoluble in most solvents, so its application is expanded when it is converted to chitosan [44]. Chitin can be converted to chitosan by an alkaline or enzymatic deacetylation process, in which the acetyl group is converted to hydroxyl (-OH) and amino (-NH_2_) groups [45]. The structures of chitin and chitosan are shown in Figure 2.

### 2.3. Unsaturated Fatty Acids

**Figure 3:**
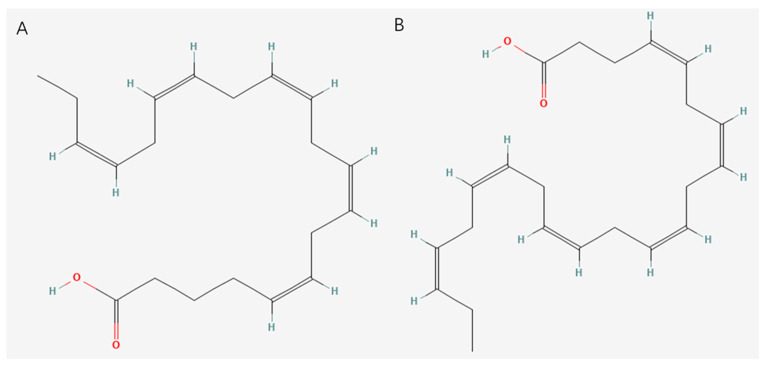
Structure diagram of (**A**) EPA and (**B**) DHA.

Unsaturated fatty acids of marine origin refer to fatty acids extracted from marine organisms that contain one or more double bonds. Compared with saturated fatty acids, there are one or more carbon double bonds in the carbon chain of unsaturated fatty acids, which give unsaturated fatty acids unique chemical properties and biological activities. Marine organisms such as fish, shellfish, seaweed, and marine micro-organisms are rich in a variety of unsaturated fatty acids, among which the most famous are omega-3 polyunsaturated fatty acids (n-3 PUFAs) [46], such as eicosapentaenoic acid (EPA) and docosahexaenoic acid (DHA), as well as monounsaturated fatty acids (MUFAs). The structures of EPA and DHA are shown in Figure 3. EPA is an n-3 PUFA that comprises a 20-carbon chain, making it a long-chain n-3 PUFA, with five cis double bonds. The double bonds can be found at carbons 5, 8, 11, 14, and 17. DHA possesses a 22-carbon chain. Its structure contains six cis double bonds located at carbons 4, 7, 10, 13, 16, and 19 [47,48].

### 2.4. Superoxide Dismutase of Marine Organisms

SOD is an important antioxidant enzyme that protects cells from oxidative damage. As a key antioxidant enzyme, SOD can effectively scavenge ROS in the body and protect cells from oxidative damage [49]. Marine SOD, derived from extremophiles like Pyrococcus furiosus, exhibits exceptional thermostability (activity retained at 90 °C) and catalytic efficiency (kcat/Km = 2.5 × 10^7^ M⁻^1^s⁻^1^), making it superior to bovine SOD (kcat/Km = 1.1 × 10^6^ M⁻^1^s⁻^1^) [50]. Compared with other sources of SOD, marine-derived superoxide dismutase has significant advantages such as natural purity, high activity, good stability, and wide sources. Marine organisms grow in a relatively pollution-free environment, making marine SOD more pure and natural, avoiding land pollution and pesticide residues. Its activity is usually higher than that of SOD from plants and animals. It can more effectively scavenge superoxide radicals in the body and play a stronger antioxidant role [51]. In addition, marine SOD can still maintain high activity under high-temperature, acid-base, and other harsh conditions, making it widely used in food, health products, cosmetics, and other fields. However, although SOD has significant antioxidant activity and a variety of potential applications, it still has some shortcomings and limitations. First of all, as a protease, SOD has low bioavailability in vivo and is easily degraded by digestive enzymes after oral administration, making it difficult to be effectively absorbed and utilized. Secondly, SOD has poor stability in conventional environments and is susceptible to temperature pH; furthermore, due to the influence of environmental factors such as light, its application scope and storage life in food, health products, and drugs are limited.

### 2.5. Vitamins

Vitamin C, found in marine sources such as seaweed and microalgae, is a powerful antioxidant that can directly scavenge free radicals in the body and reduce the damage of oxidative stress to cells. Vitamin C is abundant in red algae (*Porphyra* spp.) and microalgae (*Chlorella vulgaris*), with porphyra yezoensis containing up to 3 mg/g dry weight. And it helps protect cell membranes and Deoxyribonucleic Acid (DNA) from oxidative damage, thereby reducing the risk of a variety of oxidative stress-related diseases. A study by Yu et al. showed that prophylactic vitamin C supplementation could regulate DNA demethylation to prevent cisplatin-induced acute kidney injury in mice [52]. Vitamin E is a lipid-soluble antioxidant that can be extracted from algae such as laver and fish such as salmon and mackerel [53,54,55], and it can protect cell membrane lipids from oxidative damage. It plays an important role in the prevention of cardiovascular disease, neurodegenerative disease, and other oxidative stress-related diseases by inhibiting lipid peroxidation and maintaining the integrity and function of cell membranes [56,57]. It also helps to promote wound healing and reduce scar formation, and it has a certain repair effect on skin damage caused by oxidative stress [58].

## 3. Antioxidant Mechanism and Potential of Disease Treatment

**Figure 4:**
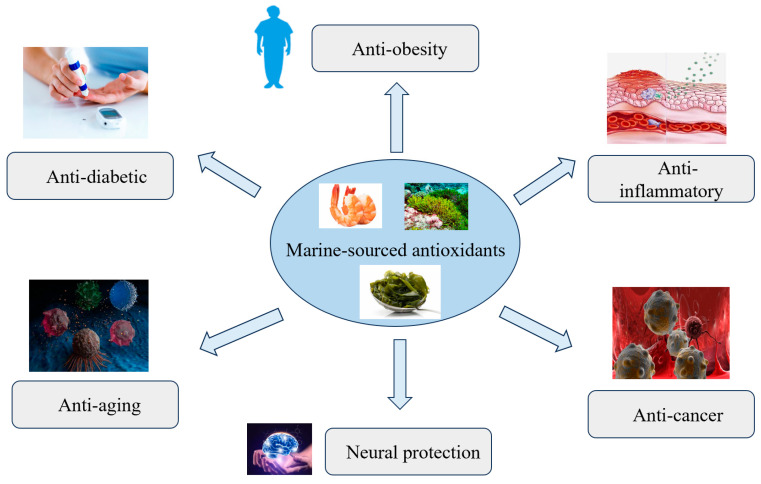
Applications of marine compounds in diseases.

Uncontrolled ROS levels can interfere with the normal activity of metabolites, such as lipids, proteins, and DNA, leading to the development of multiple diseases, as shown in Figure 4. These may include diabetes, cancer, and cardiovascular diseases. However, it has been proven that antioxidants can prevent these health problems [59,60]. Antioxidants are substances that inhibit oxidation by disrupting the chain reaction of free radicals. They provide hydrogen molecules, stabilize free radicals, and prevent them from initiating or promoting further lipid oxidation or reacting with biomolecules [61].

The effectiveness of polysaccharides as antioxidants depends on their structure, especially their degree of sulfation, molecular weight, monosaccharide content, and type of glycosidic bond. For instance, these factors may influence the ability of the compounds to provide hydrogen atoms to free radicals [62]. The antioxidant activity displayed by these compounds can generally be due to their ability to scavenge ROS or regulate the antioxidant system (i.e., the regulation of SOD, catalase (CAT), and glutathione (GSH) levels), or it can even occur through oxidative stress-mediated induced pathways (e.g., SIRT1/AMPK/PGC1α and MAPK) [14]. Fucoidan has been reported to prevent oxidation-induced kidney damage by exerting its antioxidant activity, which further inhibits the possible occurrence of kidney stones [63]. Jiang et al. showed that laminaria polysaccharide has antioxidant activity due to its ability to reduce the level of ROS formation in porcine embryos cultured in vitro [64]. In another study, a crude extract of laminaria polysaccharide from the seaweed species Laminaria had higher antioxidant capacity than the purified version. Therefore, it can be concluded that, although laminaria polysaccharide is an antioxidant, its activity depends on the species of algae and the structure of the polysaccharide [65]. Wozniak et al. obtained five sulfate fucosans from brown algae, four of which prevented the accumulation of amyloid beta and AD-like tau in Vero cells infected with herpes simplex virus type Ⅰ (HSV1). HSV1 has been reported to induce the formation of amyloid beta and abnormally phosphorylated tau (P-tau), thereby inducing the development of Alzheimer’s disease [66]. Anraku et al. evaluated the effect of a high-molecular-weight chitosan supplement (HMCS), Chitosamin^®^, on the ROS levels in 10 individuals. The researchers noticed a decrease in the levels of lipid hydroperoxides and other uremic toxins and the suppression of further stimulation of oxidative stress in the systemic circulation. Thus, this supplement presents a particular antioxidant effect, which suggests that Chitosamin^®^ can aid in the treatment of more serious illnesses such as renal failure [67]. In another study, chitin was extracted from the exoskeleton of a lobster (*Thenus unimaculatus*) shell and subsequently converted into chitosan, and its properties were evaluated. The results showed that chitosan has promising bioactivities, namely, antioxidant activity, and that it can be used in the pharmaceutical industry, as it is non-toxic [61]. Regarding alginate, a study involving the development of low-molecular-weight alginate by heat treatment showed that it had higher antioxidant activity than alginate polymers. This increase in activity is most likely due to the formation of additional functional groups. Therefore, antioxidants such as low-molecular-weight alginate may be beneficial ingredients in the food industry [68].

It is well known that n-3 PUFAs are important components of platelet phospholipid membranes. Therefore, they play a crucial role in platelet function and have been studied for their antiplatelet properties [69]. For over 20 years, people have been encouraged to supplement with n-3 polyunsaturated fatty acids to inhibit the development of cardiovascular diseases [70]. Although these supplements are taken voluntarily and prescribed for various medical conditions, they are mainly used for the primary and secondary prevention of cardiovascular diseases [71]. n-3 PUFA is able to alter cell structures and cell signaling by altering the structure of lipids within cell membranes [72]. This has been demonstrated in several animal studies, which report that adding n-3 PUFA to the diet can alter cell function [73]. The incorporation of n-3 PUFAs into the cell membrane can also modulate ion channels, such as L-type calcium (Ca^2+^) and sodium (Na^+^) [74]. Another area of mechanistic interest has been the implication that n-3 PUFAs are required for the formation of specialized pro-resolving mediators (SPMs), involved in the so-called resolution of inflammation. This is a mechanism distinct from anti-inflammatory actions [75]. It has been widely reported in animal models that n-3 polyunsaturated fatty acid-derived SPM may play a role in reducing chronic inflammation by eliminating the hypothesis of inflammation [76]. DHA and EPA exert a wide range of physiological effects, including reductions in triglycerides, heart rate, blood pressure, and platelet aggregation [77,78]. Both n-3 PUFAs also enhance arterial compliance and flow-mediated dilation while also reducing pro-inflammatory cytokines and C-reactive protein (CRP) [79]. However, the link between EPA and DHA in the modulation of inflammation lipoprotein metabolism has yet to be confirmed. Hence, currently there is no clear advantage between DHA and EPA for the modulation of lipid metabolism. However, it is likely that a combination of both may yield the most advantageous health outcomes [80].

Marine vitamins with antioxidant properties play a vital role in protecting cells from oxidative stress. Antioxidant vitamins, such as vitamins C, E, and A, protect cell integrity by neutralizing free radicals, inhibiting lipid peroxidation, regenerating oxidative antioxidants, alleviating oxidative stress, and regulating the expression of genes associated with antioxidant defense mechanisms [81,82,83]. Seaweed is rich in vitamin C, and the small differences observed between the various seaweed types suggest that vitamin C levels are generally similar among different species of seaweed, regardless of their classification [84]. Vitamin E is a fat-soluble antioxidant that protects cells from oxidative damage by neutralizing free radicals, especially in lipid membranes [85]. It works synergistically with other antioxidants such as vitamin C and is essential for maintaining cell health [86,87]. Its antioxidant properties may help reduce the risk of chronic diseases.

In addition, long-term exposure to a variety of external factors, such as physical strength, chronic light, pollution, and chemicals, as well as internal factors, such as genetics, hormonal regulation, and metabolic processes, cumulates to the complex biological phenomenon known as skin aging [88]. Internal aging occurs naturally and is characterized by reduced skin elasticity, a rough skin texture, and noticeable wrinkles. In contrast, external aging is driven by environmental factors, specifically UV light, ROS, and stress [89,90]. Marine resources are an optimistic and environmentally friendly source of unique bioactive substances for the cosmetics industry, offering promising solutions for alleviating the effects of skin aging.

Overall, we summarized the representative antioxidant compounds from marine sources and their applications in diseases, as shown in Table 1.

## 4. Advantages and Challenges

### 4.1. Advantages

Marine-sourced antioxidants are natural compounds that offer a safer alternative to synthetic antioxidants, which can be potentially toxic [92]. At the same time, we listed the differences between marine antioxidants and synthetic antioxidants in terms of their main components, bioavailability, toxicity risk, etc., in Table 2. Their natural origin makes them more acceptable for use in pharmaceuticals, nutraceuticals, and cosmetics. The marine environment has a great diversity of species and provides a rich resource of bioactive compounds. These compounds include not only common antioxidants, such as polyphenols and carotenoids, but also ocean-specific compounds, such as fucoxanthin in brown algae and astaxanthin in microalgae and crustaceans. Many marine-sourced antioxidants have a variety of biological activities, in addition to their antioxidant effects. For example, the peptides in some marine organisms are not only capable of scavenging free radicals but also have antihypertensive, immunomodulatory, and antibacterial properties. This versatility makes them of great value in the development of comprehensive therapeutic drugs. Astaxanthin, known as the “king of antioxidants”, has a stronger ability to scavenge free radicals than many other antioxidants. This high biological activity enhances their efficacy in preventing and treating oxidative stress-related diseases. With the increased awareness of sustainability, marine resources offer a renewable and sustainable option. Many marine organisms can be cultivated or harvested in an environmentally friendly manner, ensuring a continuous supply of antioxidants without depleting natural resources.

### 4.2. Challenges

Extracting and purifying antioxidants from marine organisms is technically and cost-challenging. Marine organisms often have complex structures and compositions, requiring complex methods to efficiently isolate active compounds [93,94]. In addition, these processes can involve multiple steps that increase the risk of compound degradation, which reduces the final yield. Some antioxidants have low bioavailability due to their higher molecular weight or poor solubility, limiting their effect in the human body. For example, superoxide dismutase (SOD) from marine sources has low bioavailability due to its easy degradation by digestive enzymes after oral administration. We can protect SOD from degradation, enhance its stability, and improve its bioavailability by promoting its absorption in the gastrointestinal tract through the use of nanocapsules [95]. In addition, their stability can be affected by environmental factors such as light, temperature, and pH, which can lead to reduced activity during storage and application. The biological activity of antioxidants may vary significantly depending on the source organism, extraction method, and environmental conditions. This variability makes it difficult to standardize products and ensure consistent treatment outcomes. Furthermore, although marine antioxidants are generally considered natural and safe, their sources may be affected by environmental pollution. For example, coastal brown (such as Sargassum) is prone to accumulate heavy metals (such as arsenic and cadmium) [96,97], which may pose potential risks to human health if not strictly purified. In contrast, although synthetic antioxidants such as butyl hydroxyanisole (BHA) and butyl hydroxytoluene (BHT) are cheap, they have been listed as potential carcinogens (class 2b) by the International Agency for Research on Cancer (IARC), and long-term use may cause chronic toxicity [98]. Although generally considered safe, the long-term use and potential toxicity of antioxidants still require further research. Certain compounds may have adverse effects at high doses or in specific populations. In addition, much of the research on marine-derived antioxidants has been conducted in vitro or in animal models. Conducting comprehensive clinical trials to verify their efficacy and safety in humans is critical for their approval and use in clinical settings.

## 5. Conclusions and Future Perspectives

Marine-sourced antioxidants have great potential for development in the fight against oxidative stress-related diseases. This review comprehensively describes their significant application prospects in cardiovascular diseases, neurodegenerative diseases, metabolic syndrome, and cancer. The unique structure and multifunctional properties of these antioxidants make them more advantageous than synthetic antioxidants, making them ideal candidates in the fields of medicine and nutritional supplements. However, a number of challenges still need to be addressed in order to achieve their full clinical potential. Extraction and purification processes need to be optimized to increase yields and maintain biological activity. In addition, the bioavailability and stability of marine-derived antioxidants remain critical issues that need to be addressed with innovative solutions such as nanoencapsulation technologies. In addition, conducting large-scale clinical trials is essential to verify their efficacy and safety in humans. Future research should focus on exploring new marine sources, developing sustainable extraction methods, and gaining a deeper understanding of the molecular mechanisms behind the treatment’s effects. Combining advanced biotechnological approaches, such as metabolic engineering and synthetic biology, will also help accelerate the discovery and development of novel marine-derived antioxidants. By addressing these challenges and taking full advantage of marine biodiversity, a valuable resource that has not yet been fully exploited, we will open up new therapeutic avenues for the management of oxidative stress-related diseases, thereby improving global health.

## Figures and Tables

**Table 1:** Antioxidant compounds from marine sources.

Source	Compound Category	Antioxidant Mechanism	Typical Disease Application	Possible Solutions
Brown algae	Polysaccharides	Clear ROS and activate nrf2/are pathway	AD [41], kidney injury [63]	Low molecular weight improves bioavailability
Deep-sea fish	ω-3 PUFAs	Inhibit lipid peroxidation and regulate inflammatory mediators	Cardiovascular disease, metabolic syndrome [60,91]	Improvement of water solubility by nano-emulsion
Microorganisms	Enzymes	Catalytically decompose O_2_^−^ to H_2_O_2_	Oxidative stress-related inflammation and aging [89]	Genetic engineering to improve thermal stability
Microalgae	Vitamins	Direct free radical scavenging and vitamin E regeneration	Kidney injury, skin aging [90]	Microencapsulation to prevent oxidative degradation

**Table 2:** Comparison of marine antioxidants and synthetic antioxidants.

**Group**	**Antioxidants of Marine Origin**	**Synthetic Antioxidants**
Source	Marine organisms (such as algae, animals, microorganisms)	Chemical synthesis
Main ingredients	Polysaccharides; unsaturated fatty acids; vitamins	Butyl hydroxytoluene; butyl hydroxyanisole
Bioavailability	Relatively good, such as small molecular polysaccharides and unsaturated fatty acids, which are easily absorbed	Relatively low; some synthetic antioxidants have limited absorption in vivo
Toxicity risk	Relatively low, natural source, good biocompatibility	Relatively high and may be toxic after long-term use
Cost	High extraction and purification costs	Relatively low chemical synthesis cost
Application area	Food, medicine, health products, cosmetics, and other fields	Food, plastics, rubber, and other fields as antioxidants
Clinical research stage	Some of them are in preclinical or early stages, and the research continues to deepen	The research is mature, and they are widely used

## Data Availability

Not applicable.
